# Causal inference, prediction and state estimation in sensorimotor learning

**DOI:** 10.1098/rspb.2025.1320

**Published:** 2025-08-13

**Authors:** Hyosub E. Kim, Romeo Chua, Davin Hu

**Affiliations:** ^1^ Kinesiology; Neuroscience, The University of British Columbia, Vancouver, British Columbia V6T 1Z1, Canada; ^2^ Kinesiology, The University of British Columbia, Vancouver, British Columbia V6T 1Z1, Canada

**Keywords:** motor learning, motor adaptation, causal inference, Bayesian modelling, decision-making, state estimation

## Abstract

The sensorimotor system must constantly decide which errors to learn from and which to ignore. Recent work has shown that humans are remarkably precise in parsing movement errors into internally and externally generated components for this purpose: participants automatically ignore internally generated reaching errors caused by motor noise, yet implicitly adapt to size-matched externally generated errors caused by visual perturbations. Following replication of these results with 16 neurotypical adults, we formalized our understanding of this behaviour with a novel Bayesian decision-making model. The Parsing of Internal and External Causes of Error (PIECE) model frames adaptation as a process of causal inference regarding the source of error, with the magnitude of motor corrections reflecting a combination of state estimation and the observer’s degree-of-belief that their movement was externally perturbed. Thus, PIECE challenges current computational theories that posit adaptation as a process of re-aligning the perceived hand position with the movement goal. When formally compared with three representative models of this hand-to-target alignment view, we show that only PIECE can capture the precise parsing of internal versus external errors observed. Combined, this work provides a normative explanation of how the nervous system discounts intrinsic motor noise and adapts to perturbations, keeping movements finely calibrated.

## 1. Introduction

Not all motor errors are created equal. Imagine reaching for your morning coffee, but instead of your fingers landing gracefully on the mug, they unintentionally touch down with more force than intended and tip it. This error could be the result of intrinsic motor variability (i.e. noise), the random internally generated fluctuations in movement parameters that occur even during overlearned movements like reaching. In such a case, it would be counterproductive for your motor system to recalibrate its sensorimotor mapping. Adapting to motor noise, or internally generated error (IGE), which by definition is unpredictable, would result in highly unstable behaviour and potentially amplify the magnitude of future errors even during unperturbed movements [1,2]. On the other hand, if your reach is inaccurate because of an external perturbation, such as the extra inertia from a heavy watch that you just put on, then it is critical to recalibrate your sensorimotor mapping in order to account for the added weight and keep future movements accurate and precise. The sensorimotor system is constantly tasked in this manner with deciding which errors to learn from and which to ignore.

Logically, it is clear that the motor system should not adapt to motor noise but should learn from externally generated error. What is the empirical evidence that this is actually the case? Perhaps the strongest evidence comes from a remarkable study by [3] which showed that humans are able to accurately parse total movement error into its constituent parts: the error component owing to motor noise, or internally generated error (IGE), and the error component owing to an external perturbation, or externally generated error (EGE). In their study, participants experienced small, randomized visuomotor rotations in which the cursor feedback was rotated relative to their actual hand trajectory by $\pm 2^{\circ }$ or $\pm 4^{\circ }$ on every other trial [3,4]. Despite the distribution of rotations (EGEs) being matched to baseline motor variability, participants demonstrated robust implicit (as verified in a control experiment) adaptation to only the externally generated component of the total error while effectively ignoring their IGE. That is, immediately following a perturbation trial, the subsequent adaptive response was opposite in direction and proportional to the rotation size (EGE), while also statistically independent of the IGE magnitude. Consistent with this finding, a study of saccadic eye movements also reported more robust adaptation to EGE than IGE [5], and visually clamped errors [6] as small as $1^{\circ }$ elicit robust adaptation [7]. Combined, these studies indicate that, for the motor system, the source of the error determines the implicit adaptive response.

According to standard theories of adaptation, the mismatch between predicted and actual sensory consequences of a motor command, called a ‘sensory prediction error’, is the main driver of implicit adaptation [6,8,9]. The neurophysiological underpinning of sensory predictions is the efference copy, also known as ‘corollary discharge’ [10,11], which refers to the copy of the motor command that gets sent from motor cortices to subcortical and sensory regions of the brain. Importantly, computational theories of adaptation typically assume that the sensory prediction is centred on the motor goal, or more specifically, on the explicit aiming location, i.e. the path that the end effector would travel in the absence of any IGE or adaptation [8]. However, Ranjan and Smith’s study provides compelling behavioural evidence that the sensory prediction also contains information regarding the amount of central motor noise associated with each motor command [12]. They argue that the brain generates a highly accurate sensory prediction of the hand’s actual trajectory (minus any peripheral contributions to movement error), rather than the intended trajectory to the target, which explains how the motor system is able to effectively cancel out IGE from the total error. This refined definition of sensory prediction error provides a plausible explanation of how the motor system parses total movement error, but what remains to be found is an overarching computational-level theory that explains this behaviour.

The aim of the current study was to understand the computational principles underlying such discriminative and finely tuned adaptive responses. In parallel with replication of the main results of Ranjan and Smith [3], we formalized our understanding of this behaviour by developing a novel Bayesian model, referred to as the Parsing of Internal and External Causes of Error (PIECE) model. Similar to prior Bayesian models of motor adaptation (e.g. [13]) and error detection [14], causal inference regarding whether the feedback was perturbed or not is central to the PIECE model. However, PIECE is structurally different from these models in that it assumes that, in addition to vision and proprioception, the observer also has access to an efference copy-based cue of the motor command which includes associated central motor noise [3,15]. The PIECE model conceptualizes adaptation as a process of utilizing all three cues to form a posterior belief regarding the presence (or absence) of a perturbation. This posterior serves as a weight on the observer’s estimate of the perturbation magnitude, which is inferred by combining the prior on the perturbation with the visual likelihood. The observer’s adaptive motor output is a reflection of this weighted estimate. This framework runs counter to a powerful class of computational models that frames adaptation as a process of aligning the perceived hand position with the movement goal [13,16,17]. The present study challenges such a view, as only the PIECE model could accurately capture the precise parsing of IGE and EGE observed. Our work instead supports a normative view of implicit adaptation in which prediction, perception and error correction are unified.

## 2. Results

### (a) Differential adaptation to IGE–EGE

**Figure 1.**
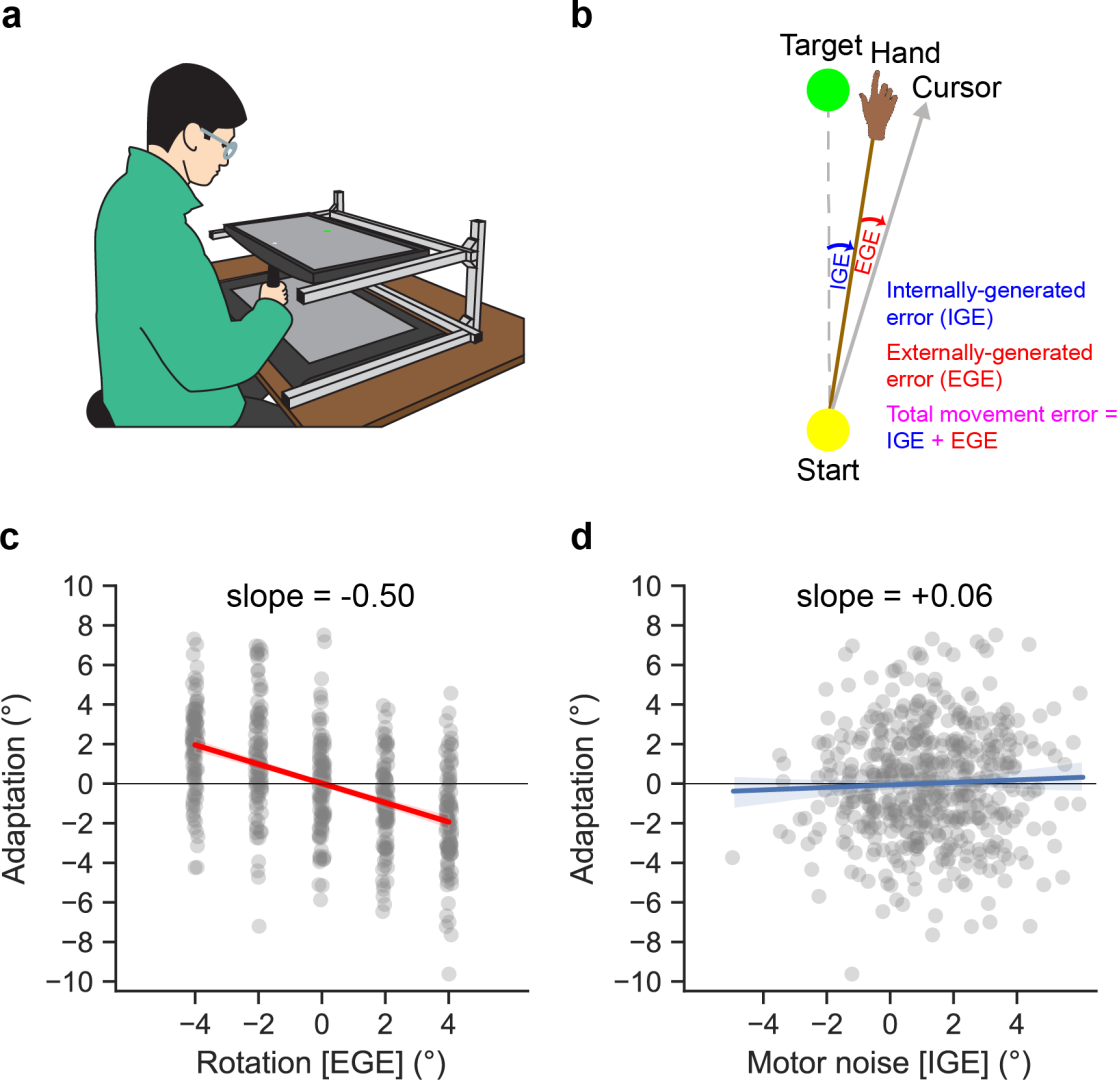
(a) Basic experimental set-up involved participants making quick point-to-point reaches by sliding a stylus along a graphics tablet. Participants could not see their hand, as experiments were conducted in a darkened room and the horizontally oriented monitor blocked vision of their hand. (b) Schematic showing how we operationally defined the two sub-components of the total error. The internally generated error (IGE) is equivalent to motor noise, or how far away from off the intended aim (i.e. the target) the reach was. The externally generated component (EGE) refers to the external perturbation—in this case, the magnitude of the visuomotor rotation. (c) Individual participant data showing adaptation as a function of EGE (grey dots represent single-trial adaptive responses). There is a clear, distinct response to EGE. (d) When the same adaptive responses are plotted as a function of IGE, there is no discernible relationship between the variables. Note that the data are shifted slightly rightwards owing to a small counter-clockwise reaching bias. The shaded regions in (c) and (d) represent bootstrapped 95% ~CIs (difficult to see owing to low variance).

We began our study by attempting to replicate the main findings of Ranjan and Smith regarding the sensorimotor system’s decomposition of total error into internally and externally generated components [3]. We tested 16 healthy, neurotypical young adults on a visuomotor task that involved fast point-to-point reaches to a visual target while controlling a small white cursor, which, depending on the trial, could be rotated by 0${,}^{\circ }$, $\pm 2^{\circ }$ or $\pm 4^{\circ }$. These rotations were pseudorandomized across the experimental block (figure 1a shows the experimental set-up) and were always surrounded by null (no rotation) trials. For our analyses, the overall motor error was decomposed into its internally and externally generated parts. The internally generated error (IGE) is defined as the random error that occurs on every reach (i.e. any angular deviation of the hand trajectory from the target), and on unperturbed reaches it, serves as the only contribution to total error. IGE is the result of intrinsic motor variability plus any intrinsic bias in reach direction [18]. Externally generated errors (EGE) are caused by external perturbations (e.g. visuomotor rotations) and are under experimental control (figure 1b). In this experiment, EGEs were defined as the visuomotor rotations, and they were therefore small and statistically independent of IGE. The intention behind utilizing such small perturbations was to keep IGE and EGE on an equal footing in terms of their magnitudes. With each perturbation trial being surrounded by null trials, adaptation was quantified as the difference in reach angle between the trial immediately following versus immediately preceding the perturbation trial: ${\text{adaptation}}_{t}={\text{hand}}_{t+1}-{\text{hand}}_{t-1}$, where t indexes the perturbation trial. Combined, these methods allowed us to easily dissociate the influence of IGE versus EGE on implicit adaptation on a trial-by-trial basis (see §5e).

In figure 1c,d we present data from an example participant. Separately plotting adaptation to EGE and IGE shows a clear dichotomy in the responses to these two different types of error. Consistent with the findings of Ranjan and Smith [3], there is robust adaptation to the externally imposed visuomotor rotations but no evidence of adaptation to spontaneously generated errors owing to motor noise, which spanned a similar range to the EGEs. This dissociation between adaptive responses to EGE versus IGE was consistent across our entire sample.

**Figure 2.**
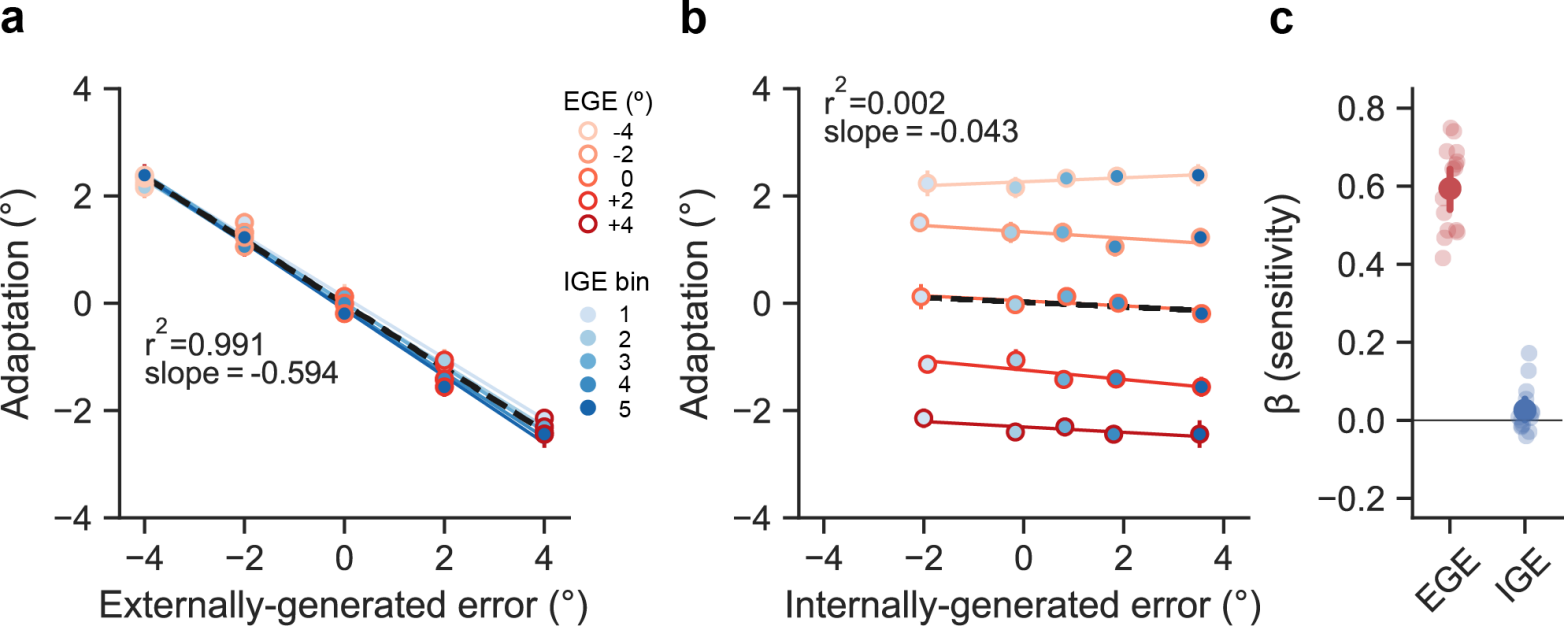
(a) Population-averaged adaptive responses were binned based on the level of EGE (shade of red) and IGE (shade of blue). The filled-in circles represent the mean of each quintile and are plotted as a function of EGE in (a), and as a function of IGE in (b) (data are shifted right owing to a small counter-clockwise reaching bias of less than 1° across participants). The vast majority of the variance in adaptive responses (99.1%) was explained by EGE. (c) Linear regression coefficients from an analysis of unbinned data (signs are flipped for ease of comparison). Error bars represent bootstrapped 95% ~CIs and are quite small in (a) and (b). More translucent dots in (c) represent individual participants.

We performed a similar set of group-level analyses to Ranjan and Smith [3] to better understand the population-averaged adaptive responses to errors. Briefly, we first binned the data based on the level of EGE, and then for each of the five levels of EGE, we further binned the data based on the level of IGE into quintiles (see §5). This procedure was applied separately for each participant. The results of these analyses are presented in figure 2a,b, where each point represents the average across every participant’s mean response for the corresponding EGE/IGE level. As seen in figure 2a, there was a clear linear response to EGE (black dashed line, mean slope = −0.594, bootstrapped 95% CI: [−0.643, −0.545], $r^{2}$= 0.991, p<0.001), with over 99% of the variance in the EGE/IGE grid being explained by the level of EGE. In stark contrast, figure 2b shows that the data were split by EGE magnitude and there was no systematic relationship between adaptation and IGE, with less than 0.2% of the variance in the EGE/IGE grid being explained by IGE (dashed black line, mean slope = −0.043 [−0.071, 0.000], $r^{2}$= 0.002, *p* = 0.82).

To further quantify the impact of EGE versus IGE on implicit adaptation, we performed a bivariate regression analysis of the unbinned data from each participant, using EGE and IGE as predictor variables. The coefficients of each predictor (sign-flipped for more convenient comparisons) were markedly different (mean difference = 0.577, 95% CI for difference scores: [0.508, 0.645]; $t_{15}=17.9,p<0.001$), reflecting the high sensitivity of each participant to EGE ($0.599[0.550,0.648],t_{15}=23.1,p<0.001$), with correspondingly low sensitivity to IGE ($0.022[-0.004,0.052];t_{15}=1.51,p=0.153$). In line with the original results of Ranjan and Smith [3], our behavioural results highlight the motor system’s clear parsing of total movement error into internally and externally generated components, effectively discounting the former and learning from the latter.

### (b) The PIECE model

**Figure 3.**
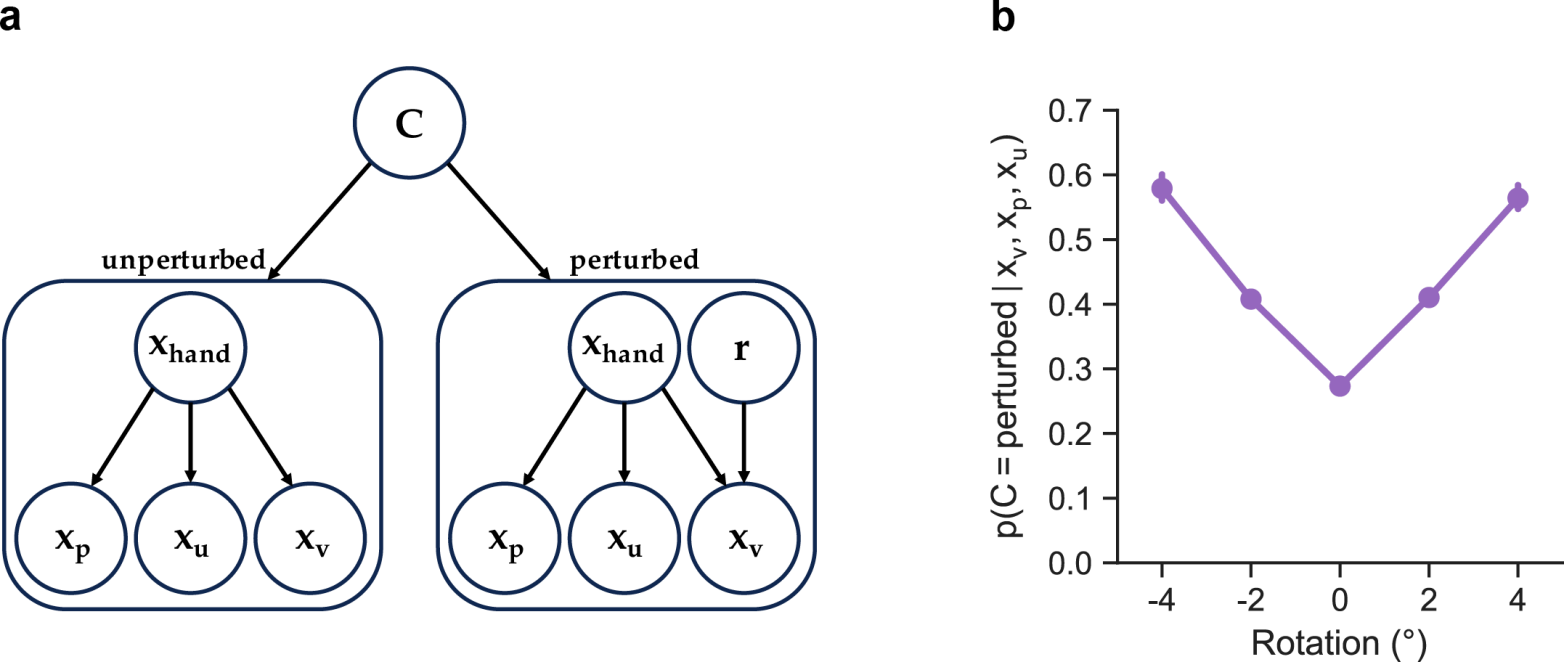
(a) Generative model for PIECE. In this model, the observer uses proprioceptive ($x_{p}$), motor predictive ($x_{u}$) and visual cues ($x_{v}$) to compute posterior probabilities of C, the causal node, which determines whether the feedback was perturbed or not. In the unperturbed case, visual feedback is a function of the actual hand position only, whereas in the perturbed case visual feedback is a function of both the actual hand position, $x_{\text{hand}}$ and the rotation, r. On a trial-by-trial basis, the posterior over C weights the observer's posterior estimate of r, which is computed by combining the prior on the rotation and the likelihood associated with the visual measurement. (b) The posterior, $p(C=\text{pert}|x_{v},x_{p},x_{u})$, as a function of the rotation size (EGE) for the same participant as shown in figure 1. This function was derived by first finding the maximum-likelihood estimates (MLEs) of this participant's data with PIECE (i.e. the parameter set that maximizes the probability of their observed data) and then using these MLEs to simulate behaviour across the experimental protocol and recording the posterior estimates.

The primary purpose of the current study was to understand the computational principles underlying the motor system’s incredibly accurate parsing of movement error into IGE and EGE. Towards this aim, we have developed a Bayesian model that optimally combines all available sensory cues in order to form a posterior belief regarding the presence (or absence) of a perturbation, with motor output reflecting the weighting of the estimated perturbation size by this degree of belief. As seen in the graphical generative model of figure 3, the observer has access to cues regarding hand position from vision ($x_{v}$), proprioception ($x_{p}$) and an efference copy-based sensory prediction of hand position ($x_{u}$), which we refer to as a ‘motor prediction’ since it stems from the motor command. The proprioceptive and motor prediction cues are unbiased estimates of the actual hand position, $x_{h}$. That is, $x_{p}∼N(x_{h},\sigma_{p}^{2})$ and $x_{u}∼N(x_{h},\sigma_{u}^{2})$. During perturbed trials with a visuomotor rotation, the visual cue is offset from the actual hand location by the size of the visuomotor rotation, $x_{v}∼N(x_{h}+r,\sigma_{v}^{2})$*,* while, by definition, r=0 during unperturbed trials. Based on a recent adaptation study that showed that participants’ visual uncertainty increases as a function of the error size, we also assume the observer’s visual uncertainty, $\sigma_{v}$, increases as a linear function of the distance of the feedback cursor from the target (i.e. the target error, e): $\sigma_{v,t}=\alpha +\beta \cdot e_{t}$, where t indexes the trial number [17].

With respect to the other nodes in the generative model, the observer’s prior on hand position, $x_{h}$, is normally distributed: $x_{h}∼N(b,\sigma_{h}^{2})$, where b represents any directional bias in reach angle relative to the target [18] (see figure 2b) and $\sigma_{h}$ is equal to the participant’s intrinsic motor variability. This prior on $x_{h}$ reflects the fact that human participants reach in a manner consistent with internal knowledge of the distribution of their intrinsic motor noise [19]. The observer also has a prior for the rotation magnitude, $p(r)∼N(0,\sigma_{r}^{2})$. We assume that the observer believes the distribution of perturbations to be normally distributed, with $\sigma_{r}$ representing the range of perturbation sizes they believe is plausible. The prior used by the observer on the causal node, which reflects their degree of belief in whether the reach was perturbed or not, matches exactly the statistics of the task and is flat: p(C=perturbed)=p(C=unperturbed)=0.5.

For our model-fitting, we calculated each participant’s motor variability during the last 50 trials of baseline reaches and used this value for $\sigma_{h}$. To further limit the number of free parameters, rather than fitting α and β of the visual uncertainty function, we used the reported mean values from the study by Zhang and colleagues (see Table S1 from [17]). In total, PIECE has three free parameters: $\sigma_{r},b$ and $\sigma_{\text{combined}}$, the latter representing the standard deviation that results from combining random variables $x_{p}$ and $x_{u}$. Briefly, while the observer may have access to separate proprioceptive and motor prediction cues, from the experimentalist’s perspective, $x_{p}$ and $x_{u}$ are not dissociable owing to their both being internal measurements centred on the true hand location. See §5g for the derivation of $\sigma_{\text{combined}}$ from $x_{p}$ and $x_{u}$.

### (c) Causal inference in PIECE

Beginning with Bayes’ Rule, the observer combines the prior, p(C), and the likelihood, $p(x_{v},x_{p},x_{u}|C)$*,* to compute the posterior for the causal node, C:


(2.1) $$\begin{array}{lrlr} & p(C|x_{v},x_{p},x_{u}) & =\frac{p(C)p(x_{v},x_{p},x_{u}|C)}{p(x_{v},x_{p},x_{u})} & \end{array}$$

As in most Bayesian cue combination models, we assume the cues are conditionally independent and their respective Gaussian distributions (likelihoods) are multiplied. Performing causal inference and computing the posterior for the unperturbed case (abbreviated as ‘¬pert’ below) requires marginalization over the hand position, $x_{h}$:


(2.2)p(C=¬pert|xv,xp,xu)∝p(C=¬pert)p(xv,xp,xu|C=¬pert)(2.3)=p(C=¬pert)∫p(xv|xh)p(xp|xh)p(xu|xh)p(xh|C=¬pert)dxh

The posterior for the perturbed case (abbreviated as ‘pert’) requires marginalization over the hand position, $x_{h}$, and the rotation magnitude, r:


(2.4)p(C=pert∣xv,xp,xu)∝p(C=pert)p(xv,xp,xu∣C=pert)(2.5)=∬p(C=pert)p(xh∣C=pert)p(r∣C=pert)p(xv∣xh,r)p(xp∣xh)p(xu∣xh)drdxh(2.6)=p(C=pert)∬p(xh∣C=pert)p(r∣C=pert)p(xv∣xh,r)p(xp∣xh)p(xu∣xh)drdxh

The denominator in equation (2.1) (i.e. the ‘marginal likelihood’) serves as a normalization factor that is common to both C=¬pert and C=pert and thus does not need to be formally computed. Instead, as a final step, we can divide the unnormalized posteriors for unperturbed and perturbed world states by their sum to normalize them.

The posterior on C defines the observer’s relative degrees of belief in both the unperturbed and perturbed world states. Figure 3b shows the posterior on C=pert as a function of the rotation size (EGE) for the same participant shown in figure 1c,d. As seen in this figure, the observer assigns more credibility to the hypothesis that their reach was perturbed as EGE increases. However, some degree of belief gets assigned to the perturbed hypothesis even during unperturbed trials, highlighting the probabilistic nature of motor adaptation [20]. In the PIECE model, these degrees of belief are combined with optimal state estimation of the perturbation magnitude.

### (d) State estimation in PIECE

The observer updates their state estimate of the perturbation with each observation (measurement). This *estimate* is denoted $\hat{r}$, whereas r represents the true perturbation, which is unknown to the observer. For this state estimation step, we assume that the observation on a given trial, $z_{t}$, which is distributed as $N(r_{t},\sigma_{v,t}^{2})$*,* is an unbiased measurement of the perturbation on that trial (i.e. the actual rotation size in degrees; [21,22]), as opposed to $x_{v,t}$*,* which is a function of the perturbation and the true hand location. Since the perturbations varied randomly in this experiment, we assumed the trial-by-trial effects were independent of each other and that there was no memory across trials [4,17]. This assumption was validated through simulation and an empirical analysis of the effect of a perturbation two trials into the future, where we observed minimal residual adaptation (electronic supplementary material, figure S1).

To update their estimate of the rotation size, the observer should combine their measurement with their prior on the rotation. Doing so leads to the proportion of the error that is corrected for on each trial being specified by $K_{t}$ in equation (2.9), where $1/\sigma_{v,t}^{2}$ and $1/\sigma_{r}^{2}$ represent the measurement and prior precisions, respectively [21,22]. The key intuition here is that the learning rate ($K_{t}$) should decrease with increasing visual uncertainty ($\sigma_{v,t}$), and conversely, increase the more volatile the observer believes the environment is, represented by a higher value of $\sigma_{r}$.

Taken together, the overall state estimate of the perturbation on trial t, ${\hat{r}}_{t}$, is a linear combination of the state estimates under the unperturbed and perturbed world state hypotheses:


(2.7) $$\begin{array}{lrlr} & {\hat{r}}_{t} & =p(C=\neg \text{pert}|x_{v,t},x_{p,t},x_{u,t}){\hat{r}}_{\neg \text{pert},t}+p(C=\text{pert}|x_{v,t},x_{p,t},x_{u,t}){\hat{r}}_{\text{pert},t} & \end{array}$$

In the unperturbed world state, there is no perturbation and so r=0 by definition. Thus, the contribution of ${\hat{r}}_{\neg \text{pert}}$ to the overall state estimate is zero. The full expression for how much the observer adapts their overall state estimate, ${\hat{r}}_{t}$, is therefore expressed as follows:


(2.8) $$\begin{array}{rl} {\hat{r}}_{t} & =p(C=\text{pert}|x_{v,t},x_{p,t},x_{u,t})\cdot K_{t}z_{t}, \end{array}$$

where


(2.9) $$\begin{array}{lrlr} & K_{t} & =\frac{\frac{1}{\sigma_{v,t}^{2}}}{\frac{1}{\sigma_{v,t}^{2}}+\frac{1}{\sigma_{r}^{2}}} & \end{array}$$

Lastly, we assume the observer’s motor output is a reflection of their estimate of the perturbation (thus opposite-signed) plus their intrinsic motor bias and motor noise:


(2.10)xh,t+1=−r^t+b+ϵt+1(2.11)ϵ∼N(0,σh2),

where $\sigma_{h}$ is measured during baseline reaches.

As expressed in equation (2.10) , the observer’s motor output reflects the combination of the estimated rotation size and the strength of their belief in the presence of an external perturbation. Thus, by combining Bayesian cue combination, causal inference and state estimation, the PIECE model unifies competing notions of the computational goal of implicit adaptation.

### (e) Model-based analyses

We evaluated the performance of four computational models of adaptation on our behavioural data: PIECE, the Proprioceptive Recalibration Model (PReMo) [16], the Perceptual Error Adaptation Model (PEA) [17] and the Relevance Estimation Model (REM) [13]. The models competing with PIECE, collectively referred to here as ‘hand-to-target alignment’ models, were chosen because of their previously demonstrated ability to explain a wide range of adaptation phenomena, the fact that they are among the most prominent Bayesian, or Bayesian-inspired, models of adaptation, and, importantly, because they each frame the computational goal of adaptation as one of aligning the perceived hand position with the movement goal. While the development of the PIECE model was informed by these other models, in stark contrast to them, PIECE posits the computational goal of adaptation as being one of correcting for the externally generated error (EGE). By objectively comparing the performance of these four models, we can adjudicate between the hand-to-target versus error correction views of adaptation.

The high-level intuition of PReMo is that mismatches between visual, proprioceptive and efference copy-based cues result in a recalibrated estimate of the ‘felt’ hand position, and implicit adaptation serves to minimize any misalignment between this perceived hand position and the motor goal, or target. PEA also posits adaptation as being driven by a perceptual error. The derivation of the perceived hand position in PEA follows optimal integration principles [23], and as mentioned earlier, the model assumes that visual uncertainty increases with error size. REM is closest in structure to PIECE, requiring a causal inference step. However, similar to PReMo and PEA, REM also assumes realignment of the estimated hand position with the target as the primary goal of adaptation.

**Figure 4.**
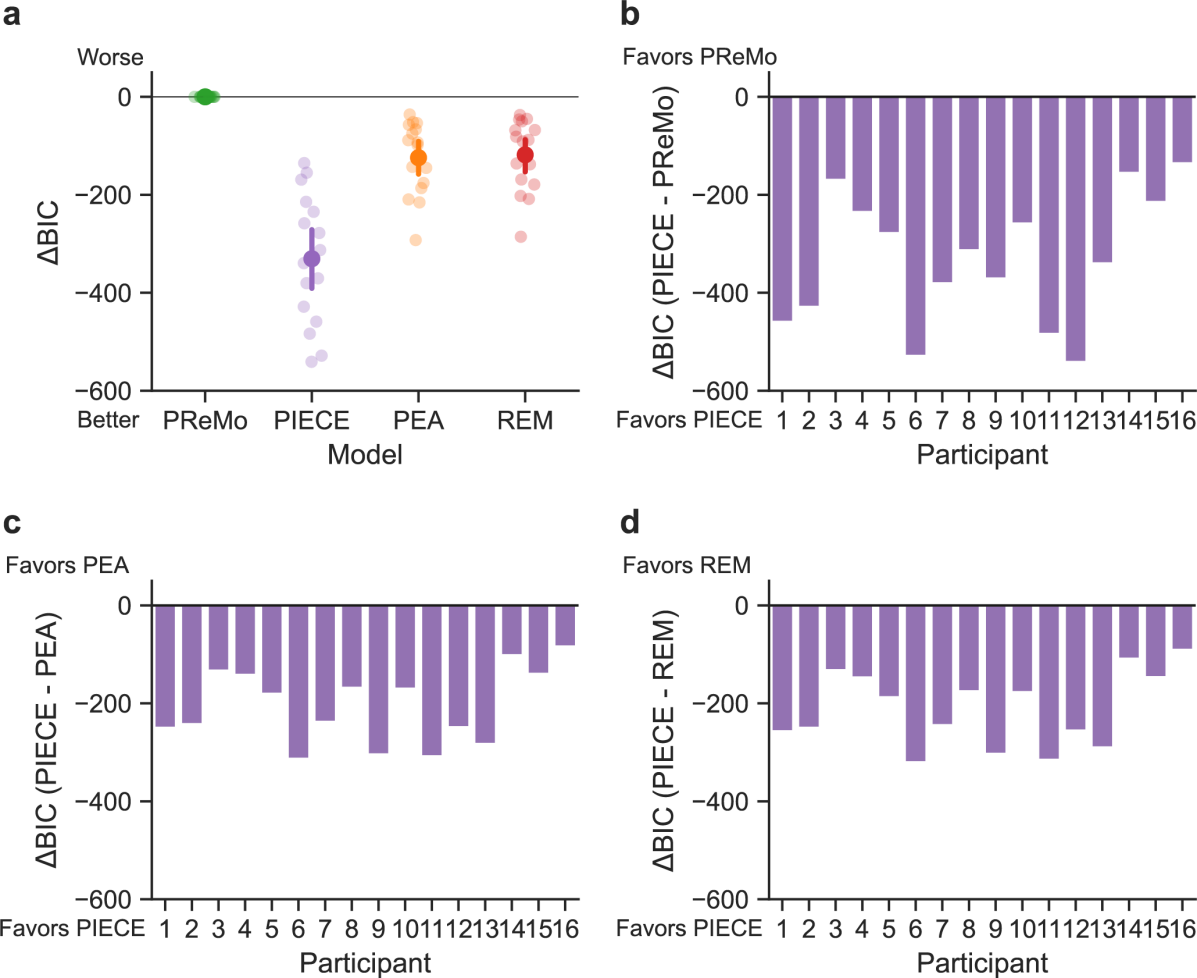
(a) Direct comparison of BIC scores of each model to PReMo’s BICs. Lower scores indicate a better model fit. (b–d) For all 16 participants, PIECE outperformed each of the other three models.

We started our analyses by using maximum likelihood estimation (MLE) to separately fit each individual’s data with all four models. After finding the set of parameters that maximized the probability of each participant’s data, we computed and compared Bayesian Information Criterion (BIC) scores. Figure 4 shows the results of this analysis, with panel (a) showing ΔBIC scores for each model when benchmarked to PReMo’s BIC scores for each individual. (We chose PReMo as the primary comparison model because it is most explicit in promoting the hand-to-target alignment idea.) Clearly, PIECE had the lowest (best) BIC scores, indicating that out of all of the models it most successfully captured the parsing of total movement error into IGE and EGE demonstrated by all participants. When directly comparing BIC scores between PIECE and each of the other three models, one can see that PIECE was not just the best overall model, but that it unanimously did the best job of fitting each individual’s data. Importantly, PIECE’s success is not an artefact of the specific paradigm used in the current study, as PEA and REM were designed, and PReMo was adapted, to fit adaptation to randomized perturbations [17].

**Figure 5.**
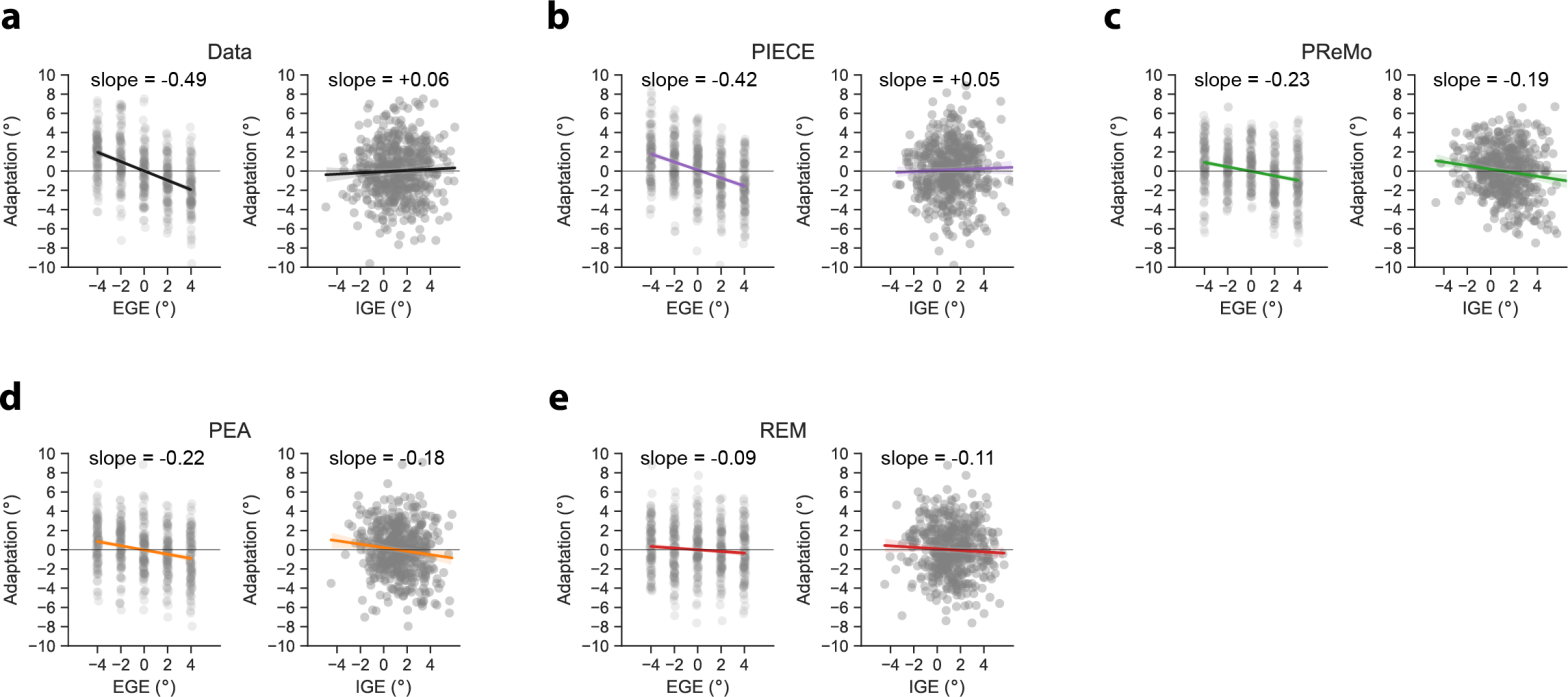
Posterior predictive check via simulation of the entire experiment using MLEs of each model’s parameter set. (a) Representative data from a single participant (P13). Adaptive responses are a linear function of EGE and remain statistically independent of IGE. (b) PIECE model mimics the empirical data: slope values indicate high sensitivity to EGE and insensitivity to IGE. (c–e) PReMo, PEA and REM all fail to capture the accurate parsing of errors into EGE and IGE. Grey dots represent adaptation measures of individual trials. Thick coloured lines and shading represent line of best fit and associated bootstrapped 95% ~CI, respectively. Posterior predictive checks of the other 15 participants followed a qualitatively similar pattern.

In addition to objective model selection criteria, we also performed a posterior predictive check of our models by generating simulated data with the best-fit parameters (see electronic supplementary material, table S1 for parameter values). The logic of this procedure is that any successful model should be able to approximate the observed data [24]. Figure 5 shows data from a representative participant, along with simulated data using each model’s max-likelihood estimates of parameter values for this participant. Only the PIECE model successfully captured the sensorimotor system’s ability to robustly adapt to small external perturbations while simultaneously discounting motor noise. Combined, we see that PIECE is the most appropriate model for these data based on objective model selection criteria, and that, qualitatively, it was the only model that could capture participants’ highly accurate decomposition of total error into constituent parts.

The behavioural data from the current study and from Ranjan and Smith [3] make clear that responses to IGE and EGE are statistically independent. For this reason, any computational framework that posits the goal of adaptation as one of aligning the hand with the target—as PReMo, PEA and REM do—is bound to fail. While those models capture adaptation to EGE, they also incorrectly predict adaptation to be driven by IGE, as clearly illustrated in figure 5. Even on unperturbed trials, the misalignment of the hand with the target owing to intrinsic motor variability will trigger an adaptive response in all models other than PIECE. However, a plethora of data now show that human participants do not treat IGE and EGE equally, which only the PIECE model was able to explain.

## 3. Discussion

As first reported by Ranjan and Smith [3] the adaptation system shows a remarkable capability to filter out IGE from total error in order to implicitly recalibrate its sensorimotor mapping in response to external perturbations [3,4]. Our successful replication of these results supports this finding and points to its robustness. To provide a computational-level explanation of the observed error parsing behaviour [25], we developed the PIECE model. PIECE is a Bayesian decision-making model that incorporates ideas of cue combination and causal inference, and takes inspiration from the three models with which it was compared in this study: PReMo, PEA and REM. By contrast to these other models, though, PIECE returns to classical ideas of adaptation as a process of state estimation with regard to the external perturbation [26]. Indeed, if one were to simply cast adaptation as a process of proportionally updating motor output by a fraction of the experienced rotation size, as in standard state-space models of adaptation [27], we could recapitulate the differential adaptation to IGE–EGE observed in our study. However, such a model does not tell us *why* or *how* the sensorimotor system decides which errors to adapt to, and which errors to ignore, not to mention other limitations of state–space models [7,26]. The unique advance offered by the PIECE model is that it provides a coherent computational-level explanation of how the sensorimotor system parses motor errors into their respective internally and externally generated components in order to ignore the former and adapt to the latter.

### (a) Implicit adaptation is not driven by perceptual error

PReMo and PEA are well-developed models that explicitly formulate the computational goal of adaptation as one of realigning the perceived hand position with the target. Both models posit that in a perturbed environment, such as a visuomotor rotation, the mismatch between sensory and motor prediction-based cues results in a proprioceptive shift towards the visual cursor, generating the driving signal for adaptation [16,17]. Although they differ in their explanation of how this shift occurs (sequential intra- and inter-modal recalibration in PReMo, Bayesian cue combination in PEA) and what constraints are placed on it, both are incapable of accommodating the error parsing observed here and first reported by Ranjan and Smith [3]. As our data and analyses make demonstrably clear, the perceived hand position, and its relationship to the target, cannot serve as the primary driver of single-trial adaptation. Figure 5 shows that, while these models can accommodate participants’ responses to EGE, they incorrectly predict adaptation in response to IGE, since the hand is by definition misaligned with the target when there is motor noise, even in the absence of a perturbation. These models also fall short because they do not incorporate causal inference.

### (b) The role of causal inference in adaptation

Our results indicate that the implicit adaptation system performs causal inference with respect to the sources of the observed total error. Bayesian causal inference models were originally developed to explain why auditory and visual cues are sometimes fused, forming a coherent percept, and why they are sometimes perceived as having two separate sources [28]. In the realm of motor adaptation, causal inference was formalized by Wei and Körding with their Relevance Estimation Model (REM) [13]. Although REM and PIECE are structurally similar in terms of causal inference, the posterior on whether the reach was perturbed or not is used in nearly opposite ways in the two models—to correct for errors that are believed to be self-generated (i.e. IGE) in the case of REM, and to correct for errors that are believed to be externally generated (i.e. EGE) in the case of the PIECE model.

According to REM, visual errors that fall within the range one should expect to observe, based on normal motor variability, are deemed more relevant and thus require an adaptive response. In their paper, the authors tested a much larger range of error sizes ($\approx 4^{\circ }-58^{\circ }$) and were not at all concerned with explaining parsing of total movement error into IGE and EGE. And similarly to nearly all adaptation studies, the methods they used did not allow them to specifically examine responses to IGE (see §5), so perhaps it should not be surprising that their proposed explanation of adaptation differs from ours. Further, REM assumes that a perturbed visual observation is owing to an ‘irrelevant’ stimulus and divorced from the actual hand position, whereas in PIECE, the observer uses the correct generative model and the visual cue is a function of hand position and rotation. Regardless of these methodological differences, the current data and the PIECE model make clear that, depending on their inferred source, size-matched errors are treated drastically differently by the motor system, and only the external component of error should be corrected for.

### (c) The role of prediction in adaptation

REM includes only proprioceptive and visual cues regarding actual hand position, while both PReMo and PEA also include an efference copy-based predictive cue of hand position. However, in PReMo and PEA the motor prediction is assumed to be centred on the motor goal (i.e. the target), rather than where the central motor system actually directs the hand, which would equal the participant’s explicit aim plus any associated IGE (and, possibly, any implicit adaptation). In this manner, the predictive cues in PEA and PReMo are akin to the prior on hand position in the PIECE model, $p(x_{h})$, which is equal to the actual distribution of unperturbed baseline reaches. In other words, the observer in PIECE possesses knowledge of their own intrinsic motor variability (and bias), an assumption made based on findings from a series of reaching studies examining rapid value-based decision making [19]. In the PIECE model, the combination of the prior on hand position and the predictive cue centred on the actual hand position, a feature lacking in the competing models, contribute to effective error parsing (see electronic supplementary material).

Based on our model fitting results, the motor prediction cue provides highly accurate and precise information regarding the actual motor command, including noise. While such accuracy may be task- and effector-specific [29], the distribution of $\sigma_{\text{combined}}$ values across our participants was quite tight, ranging from 0.2 to 1.4. This suggests that for many participants, there was very little uncertainty regarding where the hand was actually sent. These values must be interpreted with caution, however, as they actually represent the uncertainty associated with the combination of proprioceptive and motor prediction-based cues, as the two could not be dissociated in this paradigm (see §5). Regardless, these parameter estimates still suggest a highly accurate and precise prediction, as reported estimates of proprioceptive variability are generally higher than our $\sigma_{\text{combined}}$ estimates (see [17]). This is consistent with the notions of a refined sensory prediction error that incorporates IGE, and that effective parsing of small errors requires the precision of the motor prediction to be much greater than that of the movement itself [4] (electronic supplementary material, figure S2). Further, recent work has shown that even in cases of severe proprioceptive loss and during tasks that do not rely on dynamic proprioception, robust implicit adaptation is observed, likely owing to the contributions of precise motor prediction [15,30].

By contrast, noisy motor predictions, whether owing to neurological disease or injury, could have detrimental effects on error parsing and adaptation. According to PIECE simulations, depending on the combination of parameter values, impaired prediction can result in little adaptation to IGE or EGE, potentially mimicking cerebellar ataxia, or adaptation to both IGE and EGE (electronic supplementary material, figure S2). Given its flexibility in accounting for such a wide range of behaviours, PIECE affords new opportunities to provide coherent explanations of motor impairments stemming from neurological disease.

### (d) PIECE and other sensorimotor learning phenomena

PIECE incorporates a key insight from the PEA model related to goal-directed reaching—that visual uncertainty increases with error size. Zhang and colleagues empirically showed that, during reaching, the distance of the cursor from the target will increase visual uncertainty (decrease precision). An important caveat is that the authors were applying this principle to data involving implicit adaptation to visual error clamps, in which participants are explicitly instructed to ignore the cursor feedback and to fixate on the target, and the cursor trajectory is clamped to a specified angle relative to the target. Despite the difference between our tasks, our use of the same linear function for visual uncertainty is justified based on prior work which showed that the eyes automatically direct themselves towards a reaching target [31]. The success of our models further extends the findings of Zhang and colleagues, advancing the notion that whether the cursor feedback is contingent on hand position (as in our study) or not (as in a clamp study), the visual estimate of the cursor position will increase in uncertainty as a function of cursor-to-target distance.

In the Zhang *et al.* study [17], this increasing visual uncertainty is used to explain nonlinear adaptive responses to increasing perturbation sizes. Several studies have now shown that sensitivity to external perturbations is only constant over a narrow range of error size, and that adaptive responses quickly saturate as perturbation size increases [6,7,13]. Importantly, the PIECE model can also account for the saturation phenomenon. As the learning rate ($K_{t}$) in PIECE is derived from optimally combining prior and likelihood, it is a function of the observer’s prior uncertainty regarding the rotation magnitude, $\sigma_{r}$, and their visual uncertainty, $\sigma_{v,t}$. As visual uncertainty increases with rotation size, the learning rate decreases, helping to capture the saturation of adaptive responses in a principled manner and avoiding the use of an arbitrary free parameter for the learning rate as used in most state–space models. Indeed, when visual uncertainty is combined with the observer’s prior over possible perturbation sizes, PIECE can flexibly accommodate any non-monotonicities in adaptation as a function of error size (electronic supplementary material, figure S3). Also, if one were to simply add a retention parameter to PIECE (or equivalently, assume temporal dynamics in the state estimation step—see equation (2.7)), PIECE could also accommodate learning saturation and residual error during asymptotic adaptation in response to clamped feedback [6,17] and standard visuomotor rotations [32], respectively (see electronic supplementary material).

## 4. Conclusions

There are three important insights provided by the PIECE model. The first insight is that causal inference is critical for adaptation, both to infer the source of error and also to regulate how much weight to apply to the observer’s estimate of the perturbation, which in turn dictates the magnitude of single-trial adaptation. Without causal inference, other models such as PReMo and PEA are unable to capture the statistical independence between responses to IGE and EGE, since the perceptual error driving adaptation in these models is a function of hand position (IGE). Relatedly, the second insight is that accurate error parsing can only be understood through the lens of state estimation with respect to the EGE, i.e. the perturbation. Despite incorporating causal inference, the REM model also failed to account for the data because it too frames adaptation as a process of inferring the hand position for purposes of aligning it with the motor goal. However, our data and analyses make clear that adaptation responds almost exclusively to EGE. Any minimal response to IGE is attributable to the probabilistic nature of inferring and estimating the source of error—in other words, the motor system is not perfect in its error parsing. Despite this latter point, the third insight is that the internal prediction of where the hand is sent during a reach is highly accurate and precise. This was made clear in the original work of Ranjan and Smith [4], while PIECE embeds this phenomenon within a Bayesian framework. When considered within the context of high-level motor skills where the margin of error is miniscule, like playing the violin or grabbing our morning coffee in a rush, perhaps it should not be surprising that our sensorimotor system has evolved to keep accurate tabs on where it sends our limbs so that it can finely recalibrate its mapping in response to external perturbations.

## 5. Methods

### (a) Participants

Healthy, young adults were recruited from the University of British Columbia (UBC) community (*n* = 16, 8 females; average age = 22.3 years old, range: 19−29). All participants were naive to the purpose of the experiment and provided their written informed consent (consistent with the Helsinki Declaration). Participants were remunerated $15 for their participation. Experimental procedures were approved by the behavioural research ethics board at UBC under study ID H23-02324.

### (b) Experimental set-up

Participants sat in front of a horizontally oriented, 144 hertz refresh rate monitor (53.2 cm by 30 cm, ASUS), mounted directly above a graphics tablet (49.3 cm by 32.7 cm, Intuos 4XL; Wacom, Vancouver, WA), as shown in figure 1a. Participants made reaching movements while holding a stylus (power grip) embedded within a 3D-printed handle and sliding it along the graphics tablet. The stylus position was recorded at 200 Hz. On each trial, the monitor displayed a yellow start location (6 mm diameter circle) and a green circular target (6 mm diameter), with the real-time position of the stylus being indicated by a white circular cursor (3 mm diameter). Both the yellow start location and green target were positioned at the participant’s midline. The set-up and darkened testing room prevented participants from seeing their hand or arm. The experimental software was custom written using the Psychtoolbox extension in MATLAB [33].

### (c) Reaching task

Participants made quick point-to-point reaches to the straight ahead target located 9 cm from the start position. Participants initiated each trial by moving the stylus so that the white cursor entered the yellow start target. After their hand was held in the start position for at least 300 ms (hold time drawn from a uniform distribution, U[300,500]), the green target appeared 9 cm away. Participants were instructed to make quick and accurate point-to-point reaches to the target. If the movement time (elapsed time from movement onset to movement endpoint) was greater than 500 ms, the target turned red to indicate that the movement was not quick enough. Movement onset was defined as the first time point when movement velocity was ≥1 cm s^−1^ and the hand had travelled 0.5 cm from the centre of the start target. Movement end was defined as the first time point following movement onset when the velocity fell below 1 cm s^−1^ . The endpoint hand location was taken as the hand position at movement end, with frozen endpoint feedback of the cursor position provided for 500 ms. Following the completion of endpoint feedback, the yellow start target reappeared to prompt the participant to bring the cursor back to ‘home’ for the next trial.

### (d) Experimental schedule

The experiment commenced with a baseline block containing 70 null (unperturbed) trials with veridical feedback. The baseline block familiarized participants with the experimental protocol, ensuring participants made quick and accurate point-to-point reaches. Participants were informed that once they started moving, they were to ‘follow through with their reach to the end’ without correcting their movement. We were successful in ensuring participants’ reaction times (282 ms ± 40 ms; mean of medians ± SD) and movement times (365 ± 39 ms) were brisk.

To prevent the use of explicit re-aiming strategies, the experimenter provided the following instructions after the baseline block to all participants: ‘You may notice some changes to your cursor. Regardless of whether you notice these changes or not, continue to aim directly for the target and reach as quickly and accurately as possible in a straight line.’

Null and perturbation trials alternated within each experimental block. On perturbation trials involving a visuomotor rotation, the cursor was rotated away from the hand by $0^{\circ },\pm 2^{\circ }\text{or }\pm 4^{\circ }$ relative to the start position (100 trials/perturbation level).

The participants also experienced trials with no visuomotor rotation, but the target jumped by ±2°, or ±4° mid-reach (hand distance of 4.5 cm). As the focus of the current study is on visuomotor rotation results, the target jump results are provided in the electronic supplementary material.

Full visual feedback was provided during the reach as well as the return home during all visuomotor rotation trials, with the perturbation left on during both outbound and inbound portions of the trial. Consistent with [4], on half of the null trials, randomly selected, visual feedback of the cursor was completely absent during the reach. On these no visual feedback trials, following reach completion, the cursor remained hidden on the return to the start position until the hand was 1 cm from the centre of the start target. For the other 50% of null trials with visual feedback, participants received full veridical feedback following reach completion and during the return to the start position to begin the next trial. The schedule of perturbation trials was randomized.

Participants completed a total of 18 experimental blocks with 100 trials each, totalling 1800 trials. Each experimental block was separated by a minimum 1 min break.

### (e) Data analysis

All data were analysed using custom-written Python scripts and utilized standard libraries. Reach angle was defined as the angle between the straight lines connecting the start position to the target and the hand at peak velocity. This measure also quantified IGE, as we only used the straight ahead target at $0^{\circ }$. Structuring the experiment such that each perturbation trial was surrounded by a null trial on either side was critical to our analyses. Single-trial adaptation was quantified as the difference between reach angles on the post- and pre-perturbation trials (${\text{adaptation}}_{t}={\text{reach angle}}_{t+1}-{\text{reach angle}}_{t-1}$). Operationalizing adaptation this way rather than the more common method of comparing reach angles on post-perturbation to perturbation trials provides an uncontaminated measure of adaptation to internally generated motor noise. As previously explained [4], defining adaptation as ${\text{reach angle}}_{t+1}-{\text{reach angle}}_{t}$ means that the outcome measure (adaptation) contains its putative predictor, IGE (${\text{reach angle}}_{n}$), resulting in spurious correlations. We circumvent this potential confound by using the triplet analysis.

For each participant, individual trials were excluded from analysis if the reach angle had an absolute *z*-score magnitude greater than 3.5. This resulted in < 1.1% of trials being removed from any individual participant’s data, with 99.5% of all trials in the experiment being included.

We used the following binned analysis method of Ranjan and Smith (see [4]). To estimate sensitivity to EGE, trials were first split based on the level of EGE and further binned into one of five quintiles based on the magnitude of IGE (i.e. 5 levels of EGE × 5 IGE quintiles = 25 bins), separately for each participant. The average magnitude of adaptation within each of these 25 bins was then regressed onto the EGE. For estimating sensitivity to IGE, we regressed the same binned adaptation measures onto IGE. To calculate the $r^{2}$ values in figure 2, we averaged the 25 bins of adaptation versus EGE (IGE) across participants and regressed the average response onto its respective error signal.

### (f) Statistical analyses

For the analysis of unbinned data, we performed bivariate regression analysis using ordinary least-squares fitting with SciPy’s *linregress* function [34]. After confirming with QQ plots that the data could be modelled as Gaussian distributions, we compared regression coefficients with a paired *t*‐test. We also assessed whether the coefficients were reliably different from zero using a one-sample *t*‐test.

### (g) Modelling analysis

We separately fit each individual’s dataset with each of the four models using MLE with SciPy’s *minimize* function [34]. In this procedure, our goal was to find a given model’s parameter set, $\Theta_{\text{model}}$, that maximized the probability (equivalently, minimized the negative log-likelihood) of the observed trial-by-trial data. We assumed the trials were all independent of each other, and therefore summed the negative log-likelihoods across all trials. For formal model comparisons, we computed and compared Bayesian Information Criterion (BIC) scores.

To visualize how well each model could capture differential adaptation to EGE–IGE, we generated simulated data, using the actual perturbation schedule, for each participant using each model’s max-likelihood estimates of parameter values. Figure 5 shows an example ‘posterior predictive check’ for a representative participant. We note that the patterns observed for this participant were qualitatively similar across our entire sample.

Model recovery analysis with all four models and parameter recovery analysis for PIECE were performed to further validate our model fits (electronic supplementary material, figure S5; [24]).

### (h) PIECE

For the PIECE model, we note that the motor prediction, $x_{u}$, and the proprioceptive cue, $x_{p}$, are both centred on the true hand location, $x_{h}$. As we cannot dissociate them, since they are internal to the observer, we combined them, resulting in random variable $x_{\text{combined}}∼N(x_{h},\sigma_{\text{combined}})$*,* where $\sigma_{\text{combined}}=\frac{\sigma_{u}^{2}\sigma_{p}^{2}}{\sigma_{u}^{2}+\sigma_{p}^{2}}$ because $x_{\text{combined}}$ is the product $x_{u}$ and $x_{p}$, which are Gaussian.

There are three free parameters in this model: $\sigma_{\text{combined}},\sigma_{r}$ and b, the participant’s intrinsic motor bias.

### (i) PReMo, PEA and REM

Similar details regarding our fits with PReMo, PEA and REM (6, 3 and 4 free parameters, respectively) can be found in the electronic supplementary material.

## Data Availability

All data and code for this study are available at the following site: [35]. Supplementary material is available online [36].
